# Imaging the Pathophysiology of Essential Tremor—A Systematic Review

**DOI:** 10.3389/fneur.2021.680254

**Published:** 2021-06-16

**Authors:** Florian Holtbernd, N. Jon Shah

**Affiliations:** ^1^Institute of Neuroscience and Medicine (INM-4/INM-11), Forschungszentrum Juelich GmbH, Juelich, Germany; ^2^JARA-BRAIN Institute Molecular Neuroscience and Neuroimaging, Forschungszentrum Juelich GmbH, Rheinisch-Westfaelische Technische Hochschule Aachen University, Aachen, Germany; ^3^Department of Neurology, Rheinisch-Westfaelische Technische Hochschule Aachen University, Aachen, Germany

**Keywords:** essential tremor, pathophysiology, magnetic resonance imaging (MRI), tremor network, PET, SPECT, gamma-aminobutyric acid

## Abstract

**Background:** The pathophysiology underlying essential tremor (ET) still is poorly understood. Recent research suggests a pivotal role of the cerebellum in tremor genesis, and an ongoing controversy remains as to whether ET constitutes a neurodegenerative disorder. In addition, mounting evidence indicates that alterations in the gamma-aminobutyric acid neurotransmitter system are involved in ET pathophysiology. Here, we systematically review structural, functional, and metabolic neuroimaging studies and discuss current concepts of ET pathophysiology from an imaging perspective.

**Methods:** We conducted a PubMed and Scopus search from 1966 up to December 2020, entering essential tremor in combination with any of the following search terms and their corresponding abbreviations: positron emission tomography (PET), single-photon emission computed tomography (SPECT), magnetic resonance imaging (MRI), magnetic resonance spectroscopy (MRS), and gamma-aminobutyric acid (GABA).

**Results:** Altered functional connectivity in the cerebellum and cerebello-thalamico-cortical circuitry is a prevalent finding in functional imaging studies. Reports from structural imaging studies are less consistent, and there is no clear evidence for cerebellar neurodegeneration. However, diffusion tensor imaging robustly points toward microstructural cerebellar changes. Radiotracer imaging suggests that the dopaminergic axis is largely preserved in ET. Similarly, measurements of nigral iron content and neuromelanin are unremarkable in most studies; this is in contrast to Parkinson's disease (PD). PET and MRS studies provide limited evidence for cerebellar and thalamic GABAergic dysfunction.

**Conclusions:** There is robust evidence indicating that the cerebellum plays a key role within a multiple oscillator tremor network which underlies tremor genesis. However, whether cerebellar dysfunction relies on a neurodegenerative process remains unclear. Dopaminergic and iron imaging do not suggest a substantial overlap of ET with PD pathophysiology. There is limited evidence for alterations of the GABAergic neurotransmitter system in ET. The clinical, demographical, and genetic heterogeneity of ET translates into neuroimaging and likely explains the various inconsistencies reported.

## Introduction

Essential tremor (ET) is among the most common movement disorders in adulthood. Its prevalence in the general population is estimated at ~0.5% (1). ET can manifest at any age, but there is a strong association with older age, as demonstrated by a much higher prevalence (4–5%) in people aged >65 years (2). ET can manifest sporadically, but 30–70% of ET cases have a positive family history, suggesting the disease has a genetic background (3). Familial cases usually show early disease manifestation in the first two to four decades (4). The clinical hallmark of ET is a symmetric action tremor of the upper limbs (5). However, tremor may spread to other regions, such as the head, tongue, torso, jaw, and legs or can manifest as voice tremor. In some patients, signs of cerebellar impairment, such as subtle oculomotor disturbances and gait ataxia are present. Cognitive impairment and psychiatric symptoms, such as depression also can occur in ET patients (4). The term “essential tremor plus” has been coined for ET cases presenting with these additional symptoms (5). Given the heterogeneity of clinical manifestation, the variable hereditary background, and wide range of age at onset, it is likely that ET does not constitute a single disease entity, but rather a disease spectrum (4).

Despite its high prevalence, the neuronal mechanisms underpinning ET are still not fully understood. Originally, the inferior olive nucleus (ION) had been considered the central oscillator of tremor genesis in ET (6); however, this hypothesis has since been disputed, and a multiple oscillator tremor network comprising the ION, brainstem, cerebellum, thalamus, and motor cortical areas has been indicated in tremor genesis (7). Moreover, a series of histopathological studies reporting a loss and morphological alterations of cerebellar Purkinje cells gave rise to the hypothesis that cerebellar neurodegeneration may be the primary cause of ET (8–10). However, this concept has been challenged by others (11, 12). In addition, there is mounting evidence that alterations in the integrity of the inhibitory gamma-aminobutyric acid (GABA) neurotransmitter system is a contributory factor in ET pathophysiology (13). Lastly, particularly in the early course of the disease, clinical differentiation of ET from Parkinson's disease (PD) can be challenging, and some authors have suggested common pathophysiological features of the two diseases (14).

In recent decades, a substantial number of imaging techniques have emerged that enable the assessment of structural, functional, and metabolic alterations of the ET brain in a non-invasive and easily accessible way, resulting in a large body of literature. Whereas some findings corroborate with current concepts of ET pathophysiology (15–17), others do not (18, 19). More recently, novel techniques have been established to assess distinct neurotransmitter systems and their role in tremor genesis *in vivo*. Furthermore, studies exploring the dopaminergic system and cerebral iron depositions have tried to establish a connection between ET and progressive neurodegeneration, particularly with PD, providing equivocal findings (14). Here, we systematically review the advances in structural, functional, and metabolic imaging and discuss pathophysiological concepts underlying ET based on evidence from neuroimaging.

## Methods

We conducted a PubMed and Scopus search, including publications from 1966 up to December 2020, entering “essential tremor” in combination (“AND”) with any of the following terms and their corresponding abbreviations: positron emission tomography (PET), single-photon emission computed tomography (SPECT), magnetic resonance imaging (MRI), magnetic resonance spectroscopy (MRS), gamma-aminobutyric, and γ-aminobutyric acid (GABA). In addition, we browsed the reference lists of original and review articles retrieved in this primary search. We only considered articles that were (1) written in English, (2) included >5 ET subjects, (3) directly compared ET subjects with a healthy control (HC) cohort, (4) were performed on human subjects, and (5) provided quantitative or semiquantitative data analyses. We did not consider case reports, case series, or research papers that primarily focused on therapeutic interventions, such as thalamotomy, MRI-guided focused ultrasound, or deep brain stimulation (DBS). If ET patients were additionally compared with other disease groups (e.g., dystonic tremor), we solely considered comparisons with HC. We followed the PRISMA guidelines for systematic reviews (20).

We sought to address the following questions: (1) does evidence from neuroimaging support the hypothesis of cerebellar neurodegeneration in ET? (2) Do findings from neuroimaging corroborate with the postulated concept of a tremor network? (3) Is there support from neuroimaging for alterations of the GABAergic system in ET? (4) Is there evidence from neuroimaging for striatal dopaminergic degradation and nigral iron accumulation in ET as typically observed in PD?

## Results

**Figure 1:**
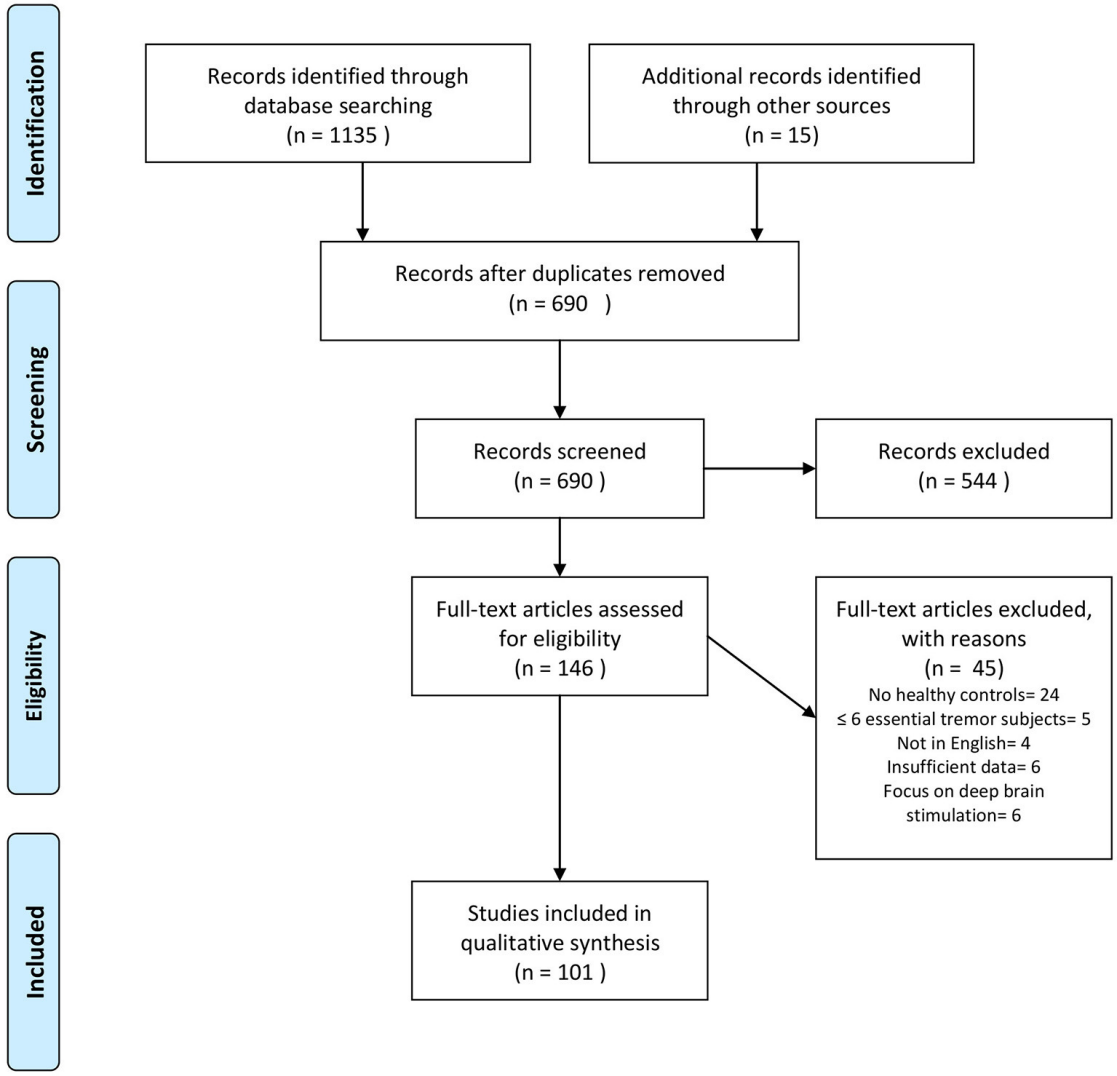
Flowchart depicting the study selection process. Search was performed in PubMed and Scopus up to December 2020. Modified from Moher et al. (20).

Our search revealed 1,135 hits. References retrieved were imported into a reference manager (Endnote X8), and duplicates were removed. FH screened all titles and abstracts for eligibility. A total of 86 papers met our inclusion criteria. Fifteen additional abstracts were identified by browsing the reference lists of papers retrieved in the database search. The senior author (JS) cross-checked papers selected for qualitative data synthesis for eligibility. A flowchart of the selection process is presented in Figure 1. Thirty-one studies were assigned to volumetric MRI, 19 to diffusion tensor imaging (DTI), 26 to functional MRI (fMRI), six to MRS, six to imaging of brain iron, three to GABAergic imaging, 17 to dopaminergic imaging, seven to perfusion imaging (PET or SPECT), and five to metabolic radiotracer imaging. Some studies applied more than one modality and were assigned to different categories accordingly. A summary of all studies included is presented in Table 1 (MRI and GABAergic imaging) and Table 2 (radiotracer imaging).

**Table 1:** Summary of MRI and GABAergic imaging studies.

**References**	**Subjects (f)**	**Age (mean ± SD)**	**Main findings**
**Volumetric MRI**			
Archer et al. (21)	ET 19 (12) HC 18 (10)	65.74 ± 11.56 63.66 ± 7.58	No difference
Bagepally et al. (22)	ET 20 (5) HC 17 (3)	38.2 ± 16.5 40.7 ± 16.5	GM of CER, frontal, occipital, L temporal, and R parietal regions ↓
Benito-Leon et al. (15)	ET 19 (10) HC 20 (10)	69.8 ± 9.4 68.9 ± 10.0	WM of R CER, L medulla, R parietal lobe, and R limbic lobe; and GM of bilateral CER, bilateral parietal lobes, R frontal lobe, and R insula ↓ No difference between hET and clET
Benito-Leon et al. (23)	13 (7) 17 (7)	67.8 ± 7.3 64.1 ± 11.9	GM (cortical thickness or volume) of both thalami, L PMC/SC, L temporal lobe, L occipital, L cingulate, bilateral entorhinal and ventral areas ↓ CER not assessed
Bhalsing et al. (24)	ET 25 (6) HC 25 (6)	45.0 ± 10.7 45.4 ± 10.7	GM of CER, R medial frontal gyrus in cognitively impaired ET patients ↓ GM of bilateral medial frontal gyrus, R SC, anterior cingulate and insula ↓ in cognitively impaired compared with cognitively intact ET group
Buijink et al. (25)	ET 36 (13) HC 30 (11)	56 ± 14 54 ± 15	No difference GM in bilateral PMC and SC, and L superior medial gyrus ↑ in hET compared with clET
Cameron et al. (26)	ET 47 (23) HC 36 (26)	76.0 ± 6.8 73.3 ± 6.5	GM of CER, posterior insula, superior temporal gyri, cingulate cortex, inferior frontal gyri, and parieto-occipital regions ↓ Pronounced atrophy in the hET subgroup
Cao et al. (27)	ET 17 (9) HC 17 (10)	39.65 ± 8.12 42.24 ± 9.47	GM of bilateral CER, occipital fusiform cortices, R inferior temporal gyrus, PMC, thalamus, midbrain, precuneus ↑ GM of L parietal lobe ↓
Cerasa et al. (28)	clET 27 (10) hET 19 (13) HC 28 (14)	65 ± 12.8 70.7 ± 7.8 66.5 ± 7.8	GM and WM of CER ↓ in hET only
Cerasa et al. (29)	ET 14 (6) HC 23 (10)	66.3 ± 9.1 64.4 ± 7.1	GM of CER in anterior lobe ↓ No difference in cerebral cortical thickness
Choi et al. (30)	ET 45 (13) HC 45 (13)	65.9 ± 6.8 67.6 ± 7.4	CER GM and WM ↓ in hET only
Daniels et al. (18)	ET 27 (9) HC 27 (9)	57.9 ± 12.2 n.a.^b^	No difference
Dyke et al. (31)	ET 47 (23) HC 36 (26)	76.0 ± 6.8 73.2 ± 6.7	GM of CER ↓ in hET and ET with voice tremor only
Espay et al. (32)	ET 16 (5)^a^ HC 25 (21)	61.7 ± 9.3 48.6 ± 11.4	GM of L CER, and occipital cortex ↓ GM of R amygdala ↑
Fang et al. (33)	ET 20 (8) HC 20 (8)	50.3 ± 14.2 50.3 ± 14.2	No difference
Fang et al. (34)	ET 35 (13) HC 35 (13)	46.86 ± 11.3 44.46 ± 11.7	No difference
Fang et al. (35)	ET 26 (7) HC 26 (7)	47.3 ± 11.3 43.4 ± 14.4	No difference
Galazzo et al. (36)	ET 10 (4) HC 10 (5)	69.4 ± 8.9 67.7 ± 7.8	GM of CER and R occipital cortex ↓
Gallea et al. (37)	ET 19 (7) HC 19 (7)	50.4 ± 15.0 50.1 ± 16.4	GM of CER ↓ GM of SMA ↑
Klein et al. (38)	ET 14 (5) HC 20 (n.a.)	61.2 ± 12.0 60.2 ± 8.1	No difference
Lin et al. (39)	ET 10 (5) HC 13 (4)	63.4 ± 8.71 65.31 ± 11.09	GM of caudate, L temporal cortex, insular cortex, L precuneus, superior temporal gyrus ↓ No difference in cerebellar volume
Nicoletti et al. (40)	ET 32 (15) HC 12 (8)	69.7 ± 9.7 67.4 ± 4.8	No difference
Novellino et al. (49)	ET 60 (32) HC 50 (25)	67.11 ± 7.84 67.58 ± 6.14	No difference in CER, thalamus, hippocampus, frontal lobe
Pelzer et al. (41)	ET 19 (9) HC 23 (8)	49.47 ± 3.51 50.93 ± 3.33	GM precuneus ↑ No difference in cerebellar volume
Pietracupa et al. (42)	ET 19 (9) HC 15 (8)	67.00 ± 17.80 63.00 ± 9.00	Thalamic volume ↑ No difference in cerebral cortical thickness or cerebellar volume
Prasad et al. (43)	ET 40 (13) HC 37 (10)	44.95 ± 12.46 46.45 ± 9.93	Cerebellar GM and volume of MCP/ICP ↓, pronounced atrophy in ET with clinical cerebellar signs No difference in WM in CER
Prasad et al. (44)	ET 40 (12) HC 40 (10)	44.95 ± 12.46 46.30 ± 9.39	GM in bilateral thalamus, hippocampus, midbrain ↓ GM in R caudate nucleus, pallidum, amygdala, bilateral putamen, nucleus accumbens ↑ CER not assessed No difference between familial vs. sporadic or between clET and rET
Qi et al. (45)	ET 27 (13) HC 27 (12)	39.65 ± 8.12 42.24 ± 9.47	GM of bilateral CER, L temporal occipital fusiform gyrus, precentral lobe, R occipital fusiform gyrus, R inferior temporal gyrus, L thalamus, midbrain, medulla, bilateral precuneus ↑ GM of L parietal lobe, pons, L insula ↓
Quattrone et al. (46)	clET 30 (12) hET 20 (14) HC 32 (16)	61.5 ± 16.5 70.6 ± 7.6^c^ 66.2 ± 8.1	GM of cerebellar anterior lobe, vermis, paravermal ↓ in hET only No difference between hET and clET
Serrano et al. (47)	ET 18 (8) HC 18 (9)	63.7 ± 10.5 63.3 ± 12.0	GM (cortical thickness or volume) in precentral, temporal, orbitofrontal, (para)hippocampal, entorhinal, posterior cingulate, and supramarginal regions ↓ CER not assessed
Shin et al. (48)	ET 39 (16) HC 36 (17)	63.7 ± 13.0 65.3 ± 6.8	GM cerebellar vermis ↓, more pronounced in clET
**Diffusion tensor imaging**			
Archer et al. (21)	ET 19 (12) HC 18 (10)	65.74 ± 11.56 63.66 ± 7.58	No difference in FA, MD not assessed
Bhalsing et al. (50)	ciET 33 (m:f 1:2.8) clET 22 (m:f 1:2.5) HC 55 (m:f 1:2.5)	47.03 ± 10.4 43.4 ± 13.4 46 ± 11	No difference in MD or FA between clET and HC MD in R cingulum and L precuneus ↑ in ciET No difference between clET and ciET
Caligiuri et al. (51)	clET 25 (14) rET 22 (11) HC 25 (11)	64.7 ± 10.9 63.7 ± 13.5 65.1 ± 6.7	Structural connectivity of thalamo-cerebello and thalamo-cortical tracts ↓ in rET and clET Structural connectivity in basal ganglia–cortical tracts ↓ in rET only
Gallea et al. (37)	ET 19 (7) HC 19 (7)	50.4 ± 15.0 50.1 ± 16.4	FA in CST ↑, no difference in tremor network-related connections
Jia et al. (52)	ET 15 (5) HC 15 (n.a.)	65.07 ± 11.41 62.07 ± 7.60	MD in red nuclei ↑, no difference in FA CER not assessed
Klein et al. (38)	ET 14 (5) HC 20 (n.a.)	61.2 ± 12.0 60.2 ± 8.1	MD in bilateral fronto-parietal and L temporo-occipital WM, and ICP ↑ FA in R ICP ↓
Martinelli et al. (19)	ET 10 (8) HC 10 (n.a.)	66 ± 11 60 ± 8	No difference in MD, FA not measured
Nestrasil et al. (53)	ET 12 (4) HC 10 (4)	45.5 ± 17.5 46.6 ± 14.8	MD in forceps minor and major, R CST, R inferior fronto-occipital fasciculi, R superior longitudinal fasciclus, R inferior longitudinal fasciculus, bilateral uncinate fasciculi, cingulum bundles, bilateral anterior thalamic radiation ↑ No FA assessment
Nicoletti et al. (54)	ET 25 (13) HC 15 (8)	62.9 ± 69.5 62.47 ± 5.4	FA in DN and SCP ↓ MD in SCP ↑
Novellino et al. (55)	ET 67 (29) HC 39 (18)	65.64 ± 10.48 64.56 ± 9.4	MD of GM in CER ↑ in ET and rET, but no difference between clET only and HC
Novellino et al. (49)	ET 60 (32) HC 50 (25)	67.11 ± 7.84 67.58 ± 6.14	MD in bilateral hippocampus, and cerebellar GM ↑
Pak et al. (56)	ET 40 (28) HC 40 (20)	44.23 ± 18.91 37.45 ± 10.95	FA in inferior longitudinal fasciculus, corpus callosum ↓ MD in inferior/superior longitudinal fasciculus, genu and corpus callosum ↑ CER not assessed
Pelzer et al. (41)	ET 19 (9) HC 23 (8)	49.47 ± 3.51 50.93 ± 3.33	MD in widespread WM including tremor network correlated with clinical tremor severity Positive correlation of callosal FA with verbal fluency test
Pietracupa et al. (42)	ET 19 (9) HC 15 (8)	67.00 ± 17.80 63.00 ± 9.00	FA ↓ and MD ↑ in multiple motor and non-motor tracts including MCP, SCP, CST, anterior thalamic radiation, longitudinal fasciculus, and inferior fronto-occipital fasciculus
Prasad et al. (57)	ET 40 (12) HC 40 (10)	44.95 ± 12.46 46.30 ± 9.39	FA in corpus callosum and CST in rET ↓ MD in CER ↑ in overall ET cohort and rET No differences of FA or MD between rET and clET
Revuelta et al. (58)	ET 18 (8) HC 10 (7)	71.1 ± 8.8 69.4 ± 9.0	MD in Vim-PMC, Vim-SMA, Vim-pre-SMA tract ↓ No difference in FA CER not assessed
Saini et al. (59)	ET 22 (5)^d^ HC 17 (3)	38.2 ± 16.5 40.7 ± 16.5	Tract-based spatial statistics whole brain: no difference in FA; MD in R internal and external capsule, and R parietal WM ↑ No difference in CER ROI based: FA in L SCP and R CST ↓ MD in right internal capsule, and left CST ↑
Shin et al. (60)	ET 10 (5) HC 8 (5)	52.8 ± 11.5 51.3 ± 11.1	FA of WM in R pons, bilateral cerebellum, L retrorubral area of the midbrain, orbitofrontal, lateral frontal, parietal, and temporal WM ↓
Tikoo et al. (61)	ET 25 (11) HC 26 (17)	68.4 ± 9.7 63.2 ± 10.3	FA ↓ and MD ↑ in cerebellar peduncles
**Functional MRI (task-based)**			
Archer et al. (21)	ET 19 (12) HC 18 (10)	65.74 ± 11.56 63.66 ± 7.58	Complex changes of activity in the tremor and visual networks during a motor task that could be modulated by increased visual feedback
Broersma et al. (62)	ET 21 (9) HC 21 (7)	51.6 ± 17.8 50.6 ± 16.4	Tremor-associated activity in L/R cerebellum, and brainstem ↑ compared with mimicked tremor in HC
Bucher et al. (16)	ET 12 (4) HC 15 (7)	61.1 ± 11.9 58.2 ± 9.8	Bilateral activation of the cerebellar hemispheres, DN, and red nuclei, and unilateral activation of the contralateral PMC/SC, thalamus, and globus pallidus in ET during involuntary tremor Higher activation of cerebellar hemispheres and red nuclei during involuntary tremor in ET compared with mimicked tremor in HC
Buijink et al. (63)	ET 31 (10) HC 29 (9)	55.4 ± 15.8 52.6 ± 16.1	Activity in CER, parietal and frontal cortex, DN and ION ↓ during motor task
Buijink et al. (64)	ET 22 (10) HC 21 (7)	59.5 ± n.a. 56.5 ± n.a.	Cerebello-motor cortical FC ↓ during motor task
Cerasa et al. (65)	ET 12 (6) HC 12 (6)	62.2 ± 12.4 59.8 ± 10.7	Activity in dorsolateral prefrontal cortex and in the inferior parietal cortex ↑ during cognitive task
Espay et al. (32)	ET 16 (5)^a^ HC 25 (21)	61.7 ± 9.3 48.6 ± 11.4	No difference during emotion processing and finger tapping task
Galazzo et al. (36)	ET 10 (4) HC 10 (5)	69.4 ± 8.9 67.7 ± 7.8	Activity in CER, sensory-motor cortex, and basal ganglia ↓ during motor task
Muthuraman et al. (66)	ET 34 (9) HC 34 (9)	58.9 ± 9 58 ± 9	Activity in CER associated with involuntary tremor mapped to motor cortex in ET, whereas it mapped to premotor cortex during mimicked tremor in HC Different topography of cerebellar activity sources in ET compared with HC
Neely et al. (67)	ET 14 (8) HC 14 (9)	61.7 ± 11.0 60.2 ± 9.2	Cerebello-cortical FC ↓ Cortico-cortical FC (PMC, SMA, premotor cortex) ↑ during motor task
Nicoletti et al. (40)	ET 32 (15) HC 12 (8)	69.7 ± 9.7 67.4 ± 4.8	Activity in CER and other nodes of the tremor network ↓ during motor task Activity in PMC and SC, precuneus and superior parietal gyrus ↑ during motor task Activity in widespread cortical regions, CER and internal globus pallidus ↑ during motor task in rET compared with clET
Passamonti et al. (68)	ET 15 (n.a.) HC 15 (n.a.)	61.6 ± 9.3 60.4 ± 7.3	FC between CER and various cortical regions implicated in focusing attention and with the DMN ↓ during cognitive task
**Functional MRI (resting state)**			
Benito-Leon et al. (69)	ET 23 (12) HC 23 (13)	63.3 ± 13.4 60.6 ± 13.2	FC in CER and visual network ↓ FC in DMN ↑
Benito-Leon et al. (70)	ET 23 (12) HC 23 (13)	63.3 ± 13.4 61.1 ± 13.1	Graph theory-based study showing complex alterations of various parameters inside and outside the tremor network in ET subjects
Fang et al. (33)	ET 20 (8) HC 20 (8)	50.3 ± 14.2 50.3 ± 14.2	Regional homogeneity in cerebellar lobes, bilateral thalamus, and the insular lobe ↓ Regional homogeneity in bilateral prefrontal and parietal cortices, L PMC, and L SMA ↑
Fang et al. (34)	ET 35 (13) HC 35 (13)	46.86 ± 11.3 44.46 ± 11.7	FC in sensorimotor network, salience network, and between anterior and posterior DMN ↑ FC in CER, and between CER and DMN and sensorimotor networks ↓
Fang et al. (35)	ET 26 (7) HC 26 (7)	47.3 ± 11.3 43.4 ± 14.4	Thalamus related FC in cerebello-thalamo-cortical network ↓ Thalamus related FC in primary and supplemental motor cortical areas ↑
Gallea et al. (37)	ET 19 (7) HC 19 (7)	50.4 ± 15.0 50.1 ± 16.4	FC between cerebellar hemispheres and ipsilateral DN, and between SMA and ipsilateral PMC ↓
Lenka et al. (71)	ET 30 (11) HC 30 (10)	45.4 ± 13.7 43.4 ± 9.2	FC of PMC and SC with R CER ↓ FC of bilateral thalamus with posterior CER ↑
Li et al. (72)	rET 20 (7) HC 27 (12)	48.32 ± 13.16 49.12 ± 11.81	Regional homogeneity in CER, putamen, and DMN ↓
Li et al. (73)	rET 19 (13) clET 31 (21) HC 25 (17)	46.58 ± 14.04 46.29 ± 14.30 49.88 ± 12.56	Activity in basal ganglia, inferior orbitofrontal gyrus, and insula ↓, activity in R CER ↑ in overall ET cohort In subgroup analysis, only clET patients showed ↑ activity in the CER Distinct differences of activity in various cortical regions and basal ganglia between rET and clET compared with HC
Mueller et al. (74)	ET 19 (4) HC 23 (n.a.)	55.5 ± 19.2 50.9 ± 18.0	Connectivity (eigenvector centrality) in cerebellar hemispheres ↓ Connectivity in the anterior cingulate and in the PMC bilaterally ↑
Nicoletti et al. (75)	ET 23 (10) HC 23 (12)	71.6 ± 10.5 70.3 ± 5.3	Complex alterations of (sensorimotor) cortico-cortical FC showing both ↓ and ↑ Cortico-cerebello FC ↓ Thalamico-cerebellar FC ↑
Tikoo et al. (61)	ET 25 (11) HC 26 (17)	68.4 ± 9.7 63.2 ± 10.3	FC of DN with L CER cortex, L caudate, L thalamus, L PMC and SC, bilateral frontal, and parietal cortices ↓
Wang et al. (76)	hET 20 (7) clET 27 (11) HC 27 (12)	51.00 ± 12.10 45.00 ± 14.43 45.00 ± 4.43	Activity in CER, bilateral caudate, R middle temporal gyrus, and L inferior parietal lobule ↑ in hET compared with HC Activity in R putamen, L precentral gyrus, and L SC ↓ in hET compared with HC Activity in thalamus, R middle temporal gyrus, R middle frontal gyrus, and R inferior parietal lobule ↑ in clET compared with HC Activity in thalamus, R middle temporal gyrus, R middle frontal gyrus, and R inferior parietal lobule ↓ in clET compared with HC
Yin et al. (77)	ET 24 (12)^e^ HC 23 (12)	46.4 ± 14.2 47.2 ± 12.8	Activity in cortical regions, mainly related to motor function (e.g., pre- and postcentral gyrus, SMA) ↑ Activity in CER ↓
**Magnetic resonance spectroscopy**			
Barbagallo et al. (78)	rET 12 (6) HC 10 (2)	69.9 ± 8.3 64.1 ± 8.3	No difference in thalamic NAA/Cr or Cho/Cr ratios
Barbagallo et al. (79)	ET 16 (3) HC 14 (4)	65.5 ± 11.1 60.8 ± 10.2	No difference in thalamic NAA/Cr or Cho/Cr ratio Thalamic Glx and Glx/Cr ratio ↑
Kendi et al. (80)	ET 14 (8) HC 9 (n.a.)	38.64 ± 12.8 35.4 ± 11.7	No difference in thalamic NAA/Cr and Cho/Cr ratios
Louis et al. (81)	ET 16 (9) HC 11 (5)	66 ± 18 60 ± 24	Cerebellar NAA/Cr ratio ↓
Louis et al. (82)	ET 20 (10) HC 11 (4)	62.2 ± 19.4 59.6 ± 23.0	No difference in cerebellar NAA/Cr ratio NAA/Cr asymmetry index between R/L cerebellar hemispheres ↓
Pagan et al. (83)	ET 10 (n.a.) HC 10 (n.a.)	59.4 ± 18.7 57.2 ± 17.0	Cerebellar NAA/Cr and Cho/Cr ratios ↓
**Imaging of brain iron**			
Cheng et al. (84)	ET 9 (n.a.) HC 166 (104)	63.8 ± 8.6^f^ 63.6 ± 6.1	No difference in nigral susceptibility-weighted imaging or nigrosome-1 integrity between ET and HC
Homayoon et al. (85)	ET 25 (10) HC 25 (12)	65.80 ± 12.82 64.60 ± 11	No difference in nigral R2* relaxation times between ET and HC
Jin et al. (86)	ET 25 (15) HC 34 (21)	61.12 ± 11.16 63.53 ± 7.81	No difference in nigral neuromelanin concentration or nigrosome-1 integrity between ET and HC
Novellino et al. (87)	ET 24 (10) HC 25 (12)	64.29 ± 10.02 64.16 ± 9.26	Higher T2* relaxation times of bilateral globus pallidus internus, substantia nigra, and R DN Only pallidal findings survived correction for multiple comparisons
Reimao et al. (88)	ET 15 (8) HC 10 (4)	70.5 ± 12.5^a^ 61.2 ± 67.3	No difference in nigral neuromelanin in ET compared with HC
Wang et al. (89)	ET 18 (7) HC 21 (11)	62.56 ± 9.31 63.52 ± 8.34	No difference in nigral neuromelanin in ET compared with HC
**Imaging of the GABAergic system**			
Boecker et al. (90)	ET 8 (4) HC 11 (6)	65.5 ± 8.0 56.6 ± 4.3	^11^C-flumazenil binding in CER, thalamus, and lateral premotor cortex ↑
Louis et al. (91)	ET 45 (19) HC 35 (25)^a^	74.98 ± 6.16 73.26 ± 6.06	No difference in DN GABA concentration between ET and HC Higher values in R compared with L DN in ET cohort, but no correlation with tremor scores
Tapper et al. (92)	ET 10 (3) HC 6 (1)	60.2 ± 9.7 62.2 ± 11.4	No difference in thalamic or CER GABA or Glx concentrations between ET and HC Positive correlation of GABA/Glx ratio with tremor severity

*ET, essential tremor; clET, classical ET; hET, ET subjects presenting with head tremor; rET, ET subjects presenting with resting tremor; HC, healthy controls; ↓, lower compared with HC; ↑, higher compared with HC; R, right; L, left; GM, gray matter; WM, white matter; CER, cerebellum; CST, corticospinal tract; DN, dentate nucleus; ICP, inferior cerebellar peduncle; MCP, middle cerebellar peduncle; PMC, primary motor cortex; SC, sensory cortex; SMA, supplementary motor area; SCP, superior cerebellar peduncle; Cr, creatine; Cho, choline; FA, fractional anisotropy; FC, functional connectivity; GABA, gamma amino-butyric acid; Glx, glutamate/glutamine; MD, mean diffusivity; NAA, N-acetylaspartate; n.a., not available*.

a *Groups not matched for gender and/or age*.

b *Age-matched, but no mean age for the cohort provided*.

c *hET significantly older than clET*.

d *Two ET subjects excluded from the final analyses because of extensive white matter lesions*.

e *Two subjects excluded due to excessive head motion*.

f *ET subjects were a subgroup of a larger cohort including atypical parkinsonian patients; no demographical data are provided for the ET group separately, but statistical analyses were performed for the ET subgroup*.

**Table 2:** Summary of radiotracer studies.

**References**	**Subjects (f)**	**Age (mean ± SD)**	**Main findings**
**Dopaminergic imaging**			
Asenbaum et al. (93)	ET 32 (19) HC 30 (20)	45 ± n.a.^a^ 63 ± n.a.	DaTScan Normal striatal uptake
Barbagallo et al. (78)	rET 12 (6) HC 10 (2)	69.9 ± 8.3 64.1 ± 8.3	DaTScan Normal striatal uptake
Benamer et al. (94)	ET 27 (9) HC 35 (20)	64.1 ± 8.8 61.1 ± 8.7	DaTScan Normal striatal uptake
Breit et al. (95)	ET 6 (4) HC 10 (5)	60 ± 5 58 ± 5	^11^Cd-threo-methylphenidate PET Normal striatal uptake
Caligiuri et al. (51)	clET 25 (14) rET 22 (11) HC 25 (11)	64.7 ± 10.9 63.7 ± 13.5 65.1 ± 6.7	DaTScan Normal striatal uptake in clET and rET
Di Giuda et al. (96)	ET 15 (9) HC 17 (10)	52.5 ± 19.5 55.3 ± 13.7	DaTScan Normal striatal uptake
Fang et al. (97)	ET 33 (23) HC 28 (10)	72.1 ± 10.0 52.3 ± 15.7	[^99m^Tc]-TRODAT SPECT Striatal uptake ↓
Gerasimu et al. (98)	ET 28 (18) HC 28 (16)	64 ± 15 63 ± 11	DaTScan Putamenal uptake ↓ No longitudinal change in 9/10 ET subjects with available follow-up scan
Isaias et al. (99)	ET 32 (10) HC 31 (18)	70 ± 7 64 ± 10	DaTScan Striatal uptake ↓
Isaias et al. (100)	ET 20 (8) HC 23 (13)	70.4 ± 9 70.5 ± 9	DaTScan Normal striatal uptake with a trend toward reductions in caudate nucleus No change over 3 years of follow-up
Lee et al. (101)	clET 9 (5) rET 6 (2) HC 21 (n.a.)	60.0 ± 11.4 68.3 ± 10.29 61.8 ± 9.7	DaTScan Normal striatal uptake in clET, ↓ in rET
Nistico et al. (102)	clET 14 (7) rET 14 (6) HC 16 (8)	68.29 ± 9.15 68.29 ± 9.15 66.37 ± 2.39	DaTScan Normal striatal uptake in clET and rET
Nistico et al. (103)	rET 10 (4) HC 20 (10)	60.60 ± 12.80 66.71 ± 4.02	DaTScan Normal striatal uptake
Novellino et al. (104)	ET 10 (6) HC 18 (9)	68.5 ± 5.13 64.06 ± 4.84	DaTScan Normal striatal uptake
Sun et al. (105)	ET 8 (n.a.) HC 11 (n.a.)	n.a.^c^ n.a.^c^	^11^C-CFT PET Normal striatal uptake
Waln et al. (106)	pET 9 (4) clET 22 (8) HC 13 (6)	67 ± 7.2 60.7 ± 8.5 63.2 ± 10.1	DaTScan Trend toward reduced striatal uptake predominantly in the caudate nucleus in both clET and ET-P
Wang et al. (107)	ET 12 (4) HC 10 (3)	52.1 ± 14.1 52.5 ± 10.7	[^99m^Tc]-TRODAT SPECT Normal striatal uptake
**Perfusion imaging**			
Boecker et al. (108)	ET 6 (4) HC 6 (n.a.)	54 ± 13.8 45 ± 18.3	${\text{H}}_{2}^{15}$O PET rCBF in bilateral CER ↑, increase diminished after intake of ethanol and was accompanied by increased rCBF of the ION
Jenkins et al. (109)	ET 11 (5) HC 8 (4)	63.8 ± n.a. 57.1 ± n.a.	C^15^O_2_ PET rCBF of bilateral CER ↑ during rest, further ↑ during involuntary tremor with additional rCBF increases of the contralateral thalamus, striatum, and PMC/SC
Wills et al. (17)	ET 7 (3) HC 6 (n.a.)	49.4 ± n.a. 51.1 ± n.a.	C^15^O_2_ PET rCBF of CER and thalamus ↑ during rest, further increase during involuntary tremor with additional increase in the red nuclei No increase in rCBF in the ION
Wills et al. (110)	ET 7 (3) HC 6 (n.a.)	49.4 ± n.a. 51.1 ± n.a.	C^15^O_2_ PET rCBF in CER, midbrain, and thalamus ↑ during involuntary tremor
Sahin et al. (111)	ET 16 (9) HC 16 (9)	29.6 ± 10 28.0 ± 7.1	Technetium-99m HMPAO SPECT No difference in rCBF, inverse correlation of frontal cortical rCBF with cognitive function
Song et al. (112)	ET 16 (7) HC 33 (23)	68.44 ± 13.73 66.94 ± 5.40	Technetium-99m HMPAO SPECT rCBF in posterior CER, frontal gyrus, cingulate, insula ↓
Song et al. (113)	clET 13 (8) hET 10 (6) HC 33 (23)	63.54 ± 20.22 65.60 ± 8.96 66.94 ± 5.40	Technetium-99m HMPAO SPECT rCBF in posterior CER, frontal gyrus, cingulate, insula ↓ No difference between clET and hET
**Metabolic imaging**			
Hallett and Dubisnky (114)	ET 8 (3) HC 10 (2)	50 ± n.a. 40 ± n.a.	FDG PET rMRG of medulla oblongata and thalamus ↑ No difference in CER
Ha et al. (115)	ET 17 (0) HC 23 (n.a.)	67.29 ± 4.79 65.35 ± 6.11	FDG PET rMRG of medial frontal lobe, medial temporal lobe, and precuneus ↓ No difference in CER
Song et al. (116)	trET 8 (0) nrET 9 (0) HC 11 (0)	65.9 ± 0.7 68.6 ± 6.4 67.2 ± 1.5	FDG PET rMRG of CER, frontal, temporal, and occipital lobes, and right precuneus ↓ rMRG of right basal ganglia ↓ in trET compared with nrET
Sun et al. (105)	ET 8 (n.a.)^b^ HC 11 (n.a.)	n.a. n.a.	FDG PET No difference in rMRG in basal ganglia, midbrain, and CER
Breit et al. (95)	ET 6 (4) HC 10 (5)	60 ± 5 58 ± 5	FDG PET No difference in rMRG in basal ganglia

*ET, essential tremor; clET, classical ET; rET, ET subjects presenting with resting tremor; pET, ET individuals presenting with one cardinal parkinsonian feature (bradykinesia, rigidity, or rest tremor); trET, ET patients responsive to propranolol therapy; nrET, ET patients unresponsive to propranolol therapy; HC, healthy controls; ↓, lower compared with HC; ↑, higher compared with HC; n.a., not available; CER, cerebellum; ION, inferior olive nucleus; PMC, primary motor cortex; SC, sensory cortex; DaT, dopamine transporter; FDG, ^18^F-fluorodeoxyglucose; PET, positron emission tomography; rCBF, regional cerebral blood flow; rMRG, regional metabolic rate of glucose; SPECT, single-photon emission computed tomography*.

a *HC significantly older than ET*.

b *Only gender and age distribution of the entire cohort are provided, and it is not clear if cohorts were matched for gender and age*.

### Structural Magnetic Resonance Imaging

A number of imaging techniques have been applied to visualize the brain morphology of ET patients *in vivo*.

#### Volumetric Imaging

Voxel-based morphometry (VBM) allows for a voxel-based automated and rater-independent analysis of brain volumes between groups, either in specified regions-of-interest (ROI) or at a whole-brain level without *a priori* hypotheses (117). Alternatively, automated segmentation methods can be applied to quantitatively measure brain volumes, e.g., the cortical thickness, using freely available software, such as FreeSurfer (118).

Cerebellar atrophy is commonly reported in ET patients (15, 22, 26, 28–32, 36, 37, 43, 46, 48). However, an equivocal number of studies found no morphological cerebellar changes (18, 21, 25, 33–35, 38–42, 49), and even increased cerebellar gray matter volume in young ET subjects has been reported (27, 45). Findings of cerebral cortical and subcortical structural changes are even more heterogeneous. There is no consistent pattern of atrophy. Moreover, alongside volume loss, gray matter volume gain has been observed in various cortical regions, and some studies did not identify any cortical differences between ET patients and HC (15, 22, 23, 26, 33–36, 38, 41, 44, 45, 47). Of note, the clinical phenotype of ET is associated with distinct morphological brain changes. For example, ET patients presenting with additional head tremor display more pronounced or distinct patterns of cerebellar atrophy, as well as various cortical structural changes compared with classic ET (25, 26, 28, 30, 31, 46). Indeed, some of the studies reporting cerebellar atrophy found significant volume loss only in ET individuals exhibiting additional head or voice tremor (28, 30, 31, 46). Moreover, cognitive dysfunction in ET has been linked to specific cortical atrophy patterns not apparent in cognitively intact ET individuals (24). The heterogeneity of structural brain alterations reported in ET has been highlighted in a recent meta-analysis including 16 VBM studies and more than 350 ET individuals (119). The latter study did not identify any brain regions, including the cerebellum, that exhibited consistent gray matter volume loss in ET patients compared with HC (119).

#### Diffusion Tensor Imaging

DTI is utilized for the assessment of the brain's microstructural integrity and is particularly sensitive to alterations in cerebral white matter. DTI measures the random movement of water molecules, which is mainly directed along white matter fiber tracts (120). Two important measures are the mean diffusivity (MD) and the fractional anisotropy (FA). The MD depicts the average movement of water molecules in organic tissue, whereas the FA refers to the directionality of movement. FA values close to 1 reflect anisotropy, whereas values nearing 0 are isotropic and are suggestive of tissue damage. Conversely, high MD values are a surrogate for a loss of cellular integrity and indicative of neuronal damage (121).

Compared with conventional MRI, DTI studies more consistently point toward microstructural alterations of the cerebellum, particularly of the cerebellar peduncles and dentate nuclei (38, 49, 54, 55, 57, 59–61). In contrast to these 11 studies, only two studies, both employing an ROI-based approach, did not find any differences in DTI between ET patients and HC (19, 21). Beyond cerebellar changes, widespread microstructural alterations have been reported in various cerebral white matter tracts related to both motor and non-motor function and in the red nuclei (37, 38, 52, 53, 56, 59). For example, in a recent study by Revuelta et al., the authors reported decreased MD of fiber tracts connecting the ventral intermediate nucleus of the thalamus (Vim), the typical target for DBS in ET, with motor and supplementary motor cortical regions (58). Even though no alterations of FA were observed in the same tracts, both MD and FA in the Vim supplementary motor area tract correlated with tremor severity, suggesting a pathological reinforcement of this tract in ET (58). Similar to VBM studies, phenotype-specific changes of FA and MD have been reported. Specifically, ET patients presenting with additional resting tremor, but unremarkable dopamine transporter imaging, showed reduced structural connectivity in a network comprised of the globus pallidus, caudate nucleus, and supplemental motor area that was not apparent in ET patients without resting tremor (51). Moreover, distinct cortical microstructural changes, including the hippocampi, have been linked to cognitive dysfunction (41, 49, 50).

### Functional and Metabolic Magnetic Resonance Imaging

The initial model of ET tremor genesis proposed that rhythmic discharges originating in the ION propagate tremor in ET (6). However, based on current research, it seems more likely that tremor genesis is not governed by a single oscillator, but is rather driven by a number of oscillators within a tremor network comprising the ION, cerebellum, thalamus, motor cortical regions, and the brainstem (7). This hypothesis is supported by evidence from neurophysiological studies confirming abnormal oscillatory activity within the tremor network in ET (122).

#### Functional Magnetic Resonance Imaging

fMRI measures the blood oxygen level-dependent (BOLD) contrast—generally called “the BOLD signal” (123). The BOLD signal is affected by hemodynamic, vascular, and metabolic factors, but is generally assumed to be closely related to neural activity (123, 124). The first task-based fMRI study in ET patients identified increased activity in the contralateral sensory and motor cortices, thalamus, and globus pallidus and bilateral overactivation of the cerebellar hemispheres and dentate nuclei during arm posturing. In contrast, the authors observed increased activity in the ION in only two out of 12 patients, supporting a pivotal role of the cerebellum in tremor genesis and refuting the single oscillator ION hypothesis (16). Subsequently, numerous task-based fMRI studies have confirmed that altered cerebellar and cerebello-thalamico-cortical activity is correlated to clinical tremor manifestation and task performance (36, 40, 62–64, 66, 67). One study did not report a significant difference in functional connectivity during an emotion processing and finger tapping task in ET patients compared with HC. However, in the latter study, HC were not age matched to the ET cohort (32). In line with the findings from structural imaging, cognitive function has been associated with specific activity changes outside the classical tremor network (65, 68).

More recently, neuronal activity has been assessed in the resting state. Resting-state fMRI is advantageous over task-based paradigms in that it is independent of individual variability in task performance and interference of tremor with motor function. The most consistent finding reported by these studies was altered intrinsic cerebellar and cerebello-thalamo-cortical activation/connectivity, particularly of cerebello-motor cortical projections (33–35, 37, 61, 71–75, 77). There is also evidence that complex functional alterations outside the classical tremor axis are present in ET, including visual networks (69, 70). In this vein, Archer et al. have demonstrated that activity within the tremor and visual networks during a grip motor task could be modulated by visual feedback (21).

Of note, surgical interventions to treat ET, such as Vim-DBS or thalamotomy have been shown to restore connectivity in the tremor network partially and to cause widespread remodeling of other brain networks outside the classical tremor axis [e.g., (125, 126)]. In line with observations from structural MRI, the clinical phenotype appears to be associated with distinct functional brain changes. For example, ET individuals exhibiting head tremor showed distinct cerebellar activity compared with those who did not (76), and ET patients with resting tremor showed different activation patterns of various cortical and subcortical brain regions compared with classical ET (73).

#### Magnetic Resonance Spectroscopy

MRS is utilized to assess neurometabolic alterations in brain tissue *in vivo*. *N*-acetylaspartate (NAA) is an abundant amino acid derivative synthesized in neurons. A reduction of NAA, therefore, is indicative of neuronal damage. The choline (Cho) fraction comprises several soluble components mainly located in myelin and cell membranes. Conditions resulting in increased turnover or damage to cellular membranes and myelin, such as inflammation, tumor, or neurodegenerative processes, result in increased Cho concentrations. Creatine (Cr) is found in most neurons and astrocytes. The Cr peak is very robust, which is why Cr is commonly used as a denominator to offset changes of NAA and Cho (127). MRS can also be applied to measure GABA (please see below).

Few studies have exploited MRS to investigate neurometabolite changes in ET. Louis et al. were the first to report a reduced NAA/Cr ratio in the cerebellum of 16 ET patients compared with 11 HC that was inversely correlated to tremor severity (81). The same group later found that NAA/Cr changes were similar between cerebellar hemispheres, in accordance with the symmetric clinical manifestation of ET (82). Similarly, reduced NAA/Cr ratios have been reported by Pagan et al. in a small cohort of 10 ET patients (83). That said, others found normal NAA/Cr ratios in the thalami of ET patients (78–80), whereas there was an increase in the excitatory neurotransmitter glutamate/glutamine evident in one of these studies (79).

#### Imaging of the GABAergic System

The role of GABA in ET has been a topic of ongoing discussion for many years (13), and different lines of research have vindicated the significance of the GABAergic system in ET pathophysiology.

ET patients show lower GABA levels in the cerebrospinal fluid (128), and GABA receptor density in the cerebellar dentate nucleus has been reported to be reduced (129). Alcohol has agonistic GABAergic properties and alleviates tremor in many patients with ET (130), and the majority of drugs used to treat ET act *via* GABAergic pathways (131). Moreover, GABA-A_1_ receptor knockout mice develop an ET-like disease that responds to GABAergic drugs commonly used to treat ET (132). The impact of reduced cerebellar GABAergic tone on neuronal activity in cerebello-thalamico-cortical tremor network activity has also been highlighted in a recent study applying a complex computational simulation model of ET (133).

Very few *in vivo* imaging studies have explored the role of GABA in ET. Using PET and ^11^C-flumazenil, a ligand of the benzodiazepine site of the GABA receptor, Boecker et al. observed increased cerebellar, thalamic, and premotor cortical tracer uptake in a small cohort of ET patients compared with HC, hinting at reduced GABAergic function (90). In contrast, a separate study employing MRS failed to demonstrate a significant difference in GABA concentration in the dentate nuclei between ET individuals and HC (91). Another MRS study reported a positive correlation of the cerebellar GABA/glutamate + glutamine ratio with clinical tremor scores in a small ET cohort. However, neither GABA nor glutamate/glutamine levels differed between ET patients and HC in the latter study (92). Given the limited number of available imaging studies focused on GABA, we would like to mention the work by Gironell et al. even though their study did not meet our inclusion criteria; they found a significant correlation of cerebellar ^11^C-flumazenil uptake and tremor severity in a cohort of 10 ET patients (134).

#### Imaging of Brain Iron

Different MRI techniques, such as susceptibility-weighted imaging, T2^*^-weighted, or its inverse R2^*^-weighted gradient echo imaging, can be used to measure brain iron (135). More recently, novel methods, such as neuromelanin and nigrosome-1 imaging have been developed to visualize the neuronal integrity of the substantia nigra (136). These techniques have been extensively used to detect iron depositions, which are assumed to be a surrogate of cellular damage in neurodegenerative disorders, such as PD (137).

In one study comparing 25 ET patients with 25 matched HC, no significant difference was found in the R2^*^ relaxation times of the substantia nigra (85). Similarly, three other studies reported normal nigral neuromelanin concentrations in ET patients (86, 88, 89), and nigral nigrosome-1 integrity has been found to be comparable with that of HC (84, 86). The focus of all these studies was on the substantia nigra, and only one study applied a whole-brain voxel-based approach (87). The authors reported increased iron levels in the bilateral pallidum, substantia nigra, and the right dentate nucleus. That being said, only the pallidal iron increase survived correction for multiple comparisons and was correlated to tremor severity (87).

### Radiotracer Imaging

Alongside MRI, PET and SPECT have been applied using a variety of tracers to study the integrity of the dopaminergic axis, brain perfusion, and metabolism in ET.

#### Dopaminergic Imaging

Epidemiological studies suggest that ET populations have an increased risk of developing PD, and there is an ongoing controversy about a potential pathophysiological overlap between the two diseases (14).

FP-CIT SPECT (commercially known as DaTScan) is commonly used to assess the presynaptic striatal dopaminergic integrity (138). Striatal tracer uptake is significantly reduced in typical and atypical parkinsonism (138).

Most studies using FP-CIT SPECT or comparable techniques found no alterations of the dopaminergic system in ET (51, 78, 93–96, 100, 102–105, 107, 139). These findings were extended by two longitudinal studies showing constant tracer uptake over time (98, 100). Of note, a third longitudinal study not meeting our inclusion criteria did not reveal a decline of striatal dopamine transporter availability over a mean follow-up period of 28 months (140). One study reported normal DaTScan in classical ET patients, whereas tracer uptake was reduced in ET patients with additional resting tremor (101). However, resting tremor ET individuals were about 7 years older than the corresponding HC, and several subjects presented with subtle parkinsonian features, raising the question of whether they may have subsequently developed PD. Conversely, others have found normal striatal dopaminergic integrity in ET patients with resting tremor (102). That being said, some authors reported signs of slight striatal dopaminergic degradation in classical ET (97–99, 106). Of note, ET patients may show reductions of tracer uptake primarily in the caudate nucleus, contrasting the typical pattern of pronounced posterior putamenal reductions observed in PD (99, 106).

#### Perfusion Imaging

A series of small exploratory PET studies conducted in the 1990s, mostly using ^15^O-labeled H_2_O and PET, revealed increased regional cerebellar blood flow (rCBF) during both rest and posture in ET patients compared with HC (17, 109, 110). These studies showed overactivation of additional regions comprising the tremor network, whereas olivary overactivation was not present in any of these studies (17, 109, 110). Boecker et al. demonstrated that abnormally increased cerebellar rCBF decreased toward normal values after ingestion of ethanol, and this decrease was correlated to concurrent tremor alleviation (108). Furthermore, there was an increase in ION activation following ethanol ingestion, suggesting increased afferent olivary input as a consequence of normalizing synaptic cerebellar activity (108). More recently, SPECT and ^99m^Tc-hexamethylpropylenaminoxom (HMPAO) have been used to measure rCBF in ET cohorts. Sahin et al. did not observe any difference of rCBF between 16 ET patients and matched HC, but reported an inverse correlation of frontal cortical rCBF with tremor severity (111). Employing the same method, Song et al. found no significant differences in rCBF between ET patients with and without head tremor (113). Interestingly, rCBF was reduced in various brain regions, including the cerebellum, in the overall ET cohort compared with HC in the latter and in a subsequent study conducted by the same group (112, 113).

#### Metabolic Imaging

^18^F-fluorodeoxyglucose (FDG) and PET can be used to quantitatively assess brain glucose consumption, which is largely equivalent to neuronal activity (141). FDG PET has been extensively used to characterize metabolic brain abnormalities in neurodegenerative disorders, such as PD, and has revealed disease-specific abnormal brain networks that correlate with disease severity and can discriminate PD from atypical parkinsonism (142).

Hallett and Dubisnky were among the first to report increased brainstem and thalamic activity in ET patients, whereas they did not observe significant changes in cerebellar metabolism (114). Similarly, two recently published studies did not find changes in cerebellar metabolism, but widespread cortical hypometabolism was reported in one of these studies (105, 115). In contrast, Song et al. found cerebellar hypometabolism accompanied by reduced tracer uptake in various cortical regions (116). In yet another study using an ROI-based approach, no metabolic differences were identified in ET patients compared with controls in the basal ganglia (95).

## Discussion

Whereas there is relatively little support from neuroimaging for the hypothesis that the ION is the primary oscillator of abnormal neuronal activity, there is robust evidence indicating that the cerebellum plays a major role in ET pathophysiology. Findings from volumetric MRI studies are, however, heterogeneous, and VBM studies do not unequivocally corroborate with histopathological findings of cerebellar neurodegeneration. Importantly, the topography of cerebellar regions displaying atrophy is inconsistent across studies, countering arguments in favor of a uniform pattern of cerebellar cell loss. DTI studies have more consistently revealed microstructural alterations of the cerebellum, and fMRI studies have clearly demonstrated abnormal cerebellar function and altered connectivity in cerebello-thalamico-cortical circuitry. Along these lines, radiotracer studies have shown increased cerebellar rCBF in ET patients, further underpinning a pivotal role of this structure in tremor genesis. That said, in view of the widespread functional alterations reported, it is likely that the cerebellum is not the sole driver of tremor genesis but rather constitutes a major hub within a multiple oscillator tremor network, thus validating neurophysiological data (122).

Findings from FDG PET studies are ambiguous. Some studies have reported extensive cortical hypometabolism, and there is evidence for increased thalamic activity. However, other studies have reported opposing results, and in particular, there are conflicting findings with respect to cerebellar metabolic activity. Data from MRS studies are scarce and insufficient to draw firm conclusions. However, the few available studies provide some evidence for thalamic and cerebellar neuronal damage.

The dopaminergic axis appears to be largely preserved in ET. This is also illustrated by the fact that DaTScan has been certified for use in the differentiation of PD from ET by both the European Medicines Agency (EMA) and the United States Food and Drug Administration (FDA), and some studies on PD even use ET cohorts as “normal” controls [e.g., (143)]. That being said, some authors have suggested slightly reduced striatal dopaminergic integrity in ET subjects that does not, however, seem to decline over time.

With the exception of one study, there is no evidence for pathological nigral iron accumulation as typically observed in PD. However, only one study did not apply an ROI-based approach limited to the substantia nigra, and this study did observe a significant increase of iron accumulation in the bilateral globus pallidus internus. Therefore, it seems that there is no relevant nigral iron accumulation in ET, but this could well be the case for other brain regions not commonly assessed by imaging studies thus far, arguing in favor of neurodegenerative processes.

Finally, MRS and radiotracer studies lend some support to the hypothesis that dysfunction of the GABAergic system is involved in ET pathophysiology. It remains to be elucidated whether the reduced GABAergic tone is a primary contributing factor to ET pathophysiology or rather a consequence of disturbed Purkinje cell function or even cell death.

**Figure 2:**
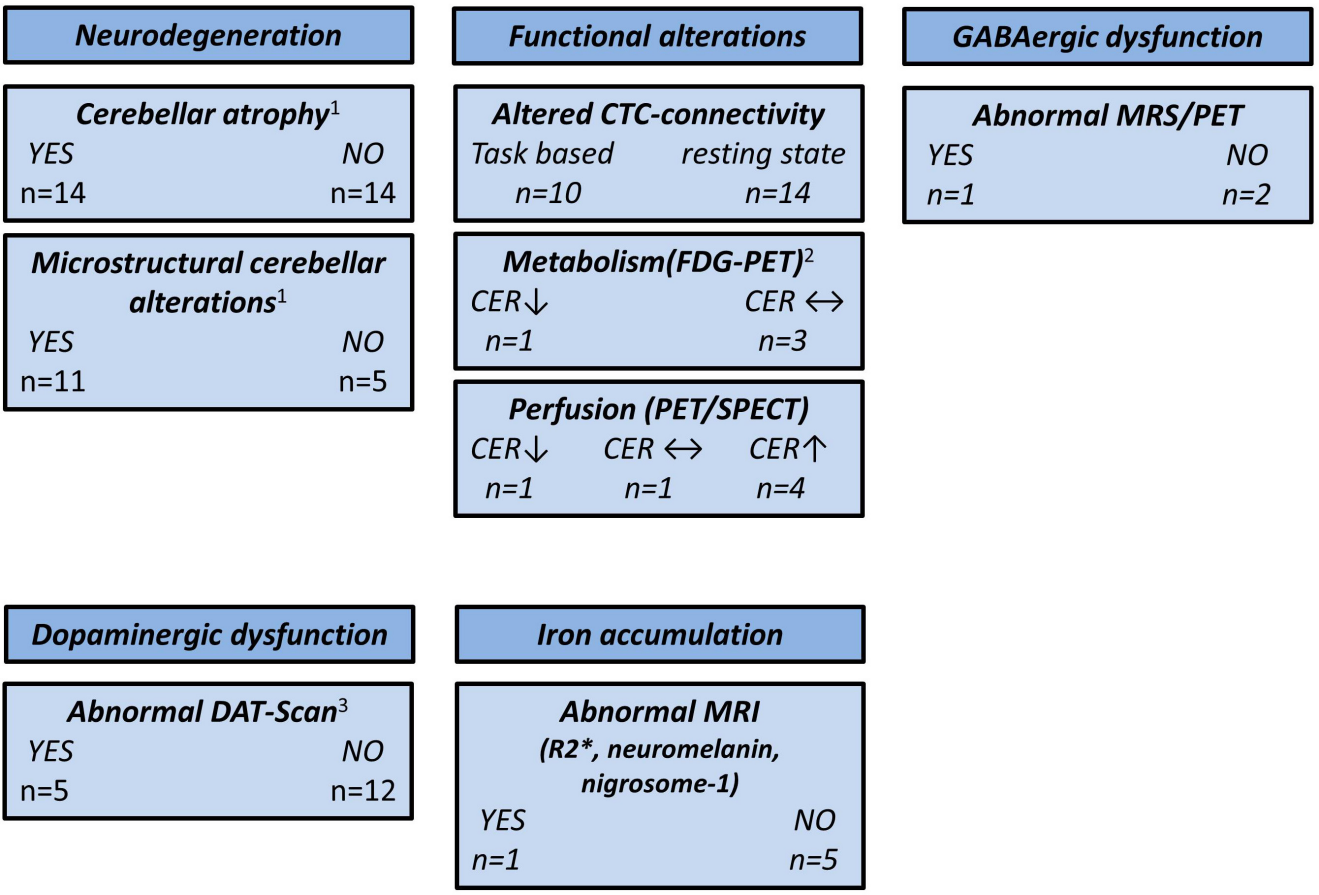
Overview of neuroimaging studies in essential tremor. CER, cerebellum; CTC, cerebello-thalamico-cortical; DAT, dopamine transporter; FDG, ^18^F-fluorodeoxyglucose; GABA, γ-aminobutyric acid; MRS, magnetic resonance spectroscopy; MRI, magnetic resonance imaging; PET, positron emission tomography; SPECT, single-photon emission computed tomography; ↑, higher compared with the healthy controls; ↓, lower compared with the healthy controls; ↔, no difference compared with the healthy controls. ^1^CER not assessed by three studies. ^2^CER not assessed by one study. ^3^One study used ^11^C-CFT PET and two studies used [^99m^Tc]-TRODAT SPECT.

Based on the epidemiological, genetic, and clinical heterogeneity, it is likely that no single ET entity exists, but rather an ET spectrum. This is supported by the notion that clinical phenotype, e.g., the distribution of tremor manifestation, the presence of cognitive impairment, or resting tremor, is linked to specific functional and structural brain changes. A summary of the main findings reported in this review is depicted in Figure 2.

Taking genetic background (familiar vs. sporadic), age at onset, disease duration, therapeutic responsiveness, and clinical phenotype (e.g., presence of head tremor, symptoms of “ET plus”) into account is important when studying ET populations, but these factors have not been consistently implemented in study designs so far. There are additional issues likely contributing to the heterogeneous findings from neuroimaging studies, such as limited sample size (this is particularly true for PET and SPECT studies), subject age (the mean age of ET cohorts included in this review ranged from 28 to 74 years), nature of the analytical approach (e.g., ROI-based vs. whole-brain analysis, statistical threshold applied), and the different field strengths of the MRI scanners. Moreover, several groups have published multiple papers on related topics using similar cohorts or did not specify if there was an overlap of cohorts across their studies, a potential source of reporting bias.

It is desirable that future studies more rigorously focus on the demographical, genetic, and clinical heterogeneity of ET. Multimodal imaging, including the simultaneous assessments of MRI, PET, and electroencephalography, may shed further light on the complex neuronal alterations underlying ET. Furthermore, the much higher spatial resolution of ultra-high field MRI may enable researchers to solve the remaining controversy of whether cerebellar neurodegeneration is the pathological foundation of ET.

## Data Availability Statement

The original contributions presented in the study are included in the article/supplementary material, further inquiries can be directed to the corresponding author/s.

## Author Contributions

FH: study design, data collection and analysis, and drafting of the manuscript. NS: study design, revision of the manuscript, and supervision. Both authors contributed to the article and approved the submitted version.

## Conflict of Interest

The authors declare that the research was conducted in the absence of any commercial or financial relationships that could be construed as a potential conflict of interest.
